# “Keep It Short and Simple”: Perceptions of patients and healthcare professionals on the use of a mobile health app in the care for patients undergoing radical prostatectomy

**DOI:** 10.1002/bco2.270

**Published:** 2023-07-15

**Authors:** Andries Clinckaert, Lucas Schreurs, Lars Wouters, Wouter Everaerts, Diederik De Cock

**Affiliations:** ^1^ Department of Urology University Hospitals Leuven Leuven Belgium; ^2^ Department of Public Health, Biostatistics and Medical Informatics Research Group Vrije Universiteit Brussel Brussels Belgium; ^3^ Department of Cellular and Molecular Medicine KU Leuven Leuven Belgium

**Keywords:** mHealth, mHealth app, prostate cancer, qualitative research, radical prostatectomy, urology

## Abstract

**Background:**

Patients undergoing radical prostatectomy for localised prostate cancer generally have good long‐term survival rates. However, late recurrences can occur and require lifelong follow‐up.

**Objective:**

This research aims to investigate different stakeholders' perceptions on the use of mobile health (mHealth) applications for prostate cancer follow‐up after radical prostatectomy.

**Methods:**

A cross‐sectional qualitative study was conducted to explore stakeholders' perceptions of an mHealth application for follow‐up after radical prostatectomy. Urologists, nurses, and patients treated with radical prostatectomy were interviewed, and data were transcribed and analysed using thematic analysis according to Qualitative Analysis Guide of Leuven. Recommended features for an ideal mHealth application were grouped according to the Persuasive Systems Design model.

**Results and Limitations:**

A total of 30 stakeholders, consisting of nurse specialists (*n* = 7), urologists (*n* = 8), and patients (*n* = 15), were interviewed. Expected benefits and barriers were mentioned and grouped in five overarching themes: healthcare optimisation, disease management, app compliance, legal and organisational requirements, and patient–mHealth interaction. Stakeholders provided a multitude of suggestions for an ideal mHealth app. Yet, not all types of stakeholders were interviewed, and patient selection limited generalisability of findings.

**Conclusions:**

Stakeholders indicate that an mHealth app in the care for post‐prostatectomy patients can improve patient care and promote disease management but consider app compliance as a major challenge.

**Patient Summary:**

We interviewed patients, nurses, and urologists about using an mHealth application for follow‐up after radical prostatectomy. The participants agreed that an mHealth app could improve care optimisation and disease management, but some concerns and barriers were expressed. This resulted in a list of recommended features for an ideal app.

## 1 INTRODUCTION

Patients with prostate cancer (PC) undergoing radical prostatectomy generally have favourable long‐term outcomes with good disease specific survival. However, depending on the tumour characteristics, a substantial proportion of patients will experience disease recurrence requiring additional therapy.^1^ Moreover, post‐prostatectomy patients can experience urinary and sexual dysfunction, requiring adequate follow‐up. The long term‐survivorship of PC patients and the need for lifelong follow‐up impose a substantial burden on healthcare services.^2^, ^3^, ^4^ Moreover, current follow‐up practices are failing to meet PC survivors needs in terms of urological, sexual, and psychosocial care.^5^

Mobile health (mHealth) applications are being used more frequently. Around 1% of all apps available on the Android and iOS platforms are focused on mHealth. mHealth has the potential to monitor post‐prostatectomy patients' health status by collecting and evaluating patient‐reported outcomes and serum biomarkers like prostate‐specific antigen (PSA).^6^, ^7^ Such an mHealth application might help to tailor post‐operative follow‐up, reducing the number of unnecessary clinic visits for asymptomatic patients with undetectable PSA while signalling the need for additional clinic visits in patients with biochemical recurrence or increasing symptoms.^4^ Moreover, in a scoping review by Pham et al.,^8^ PC was among the most targeted cancers for virtual care models, that is, any remote interaction between patients and healthcare providers using technology in cancer survivors. Hence, mHealth seems highly relevant for following‐up patients with PC. A study from 2015 reported on 150 available applications that guide the treatment of genitourinary tract diseases, with a few dedicated to prostate disease management.^9^

The perceptions of stakeholders, including patients and healthcare providers, are crucial to successfully implement an mHealth application in the follow‐up of patients with PC after surgery.^10^ Previous studies showed that patients see advantages in mHealth, including improved communication with healthcare providers, more reliable disease‐specific information, and reinforced social support.^11^, ^12^, ^13^, ^14^ However, patients and other stakeholders have also raised concerns about low user engagement, lack of scientific evidence, lack of integration into healthcare systems, lack of regulatory and privacy oversight, and lack of reimbursement for mHealth.^15^, ^16^, ^17^, ^18^

In this study, we performed semistructured interviews from different stakeholders in PC care, to explore the perceptions of patients, nurses, and urologists on the potential benefits, barriers, and essential features of an mHealth application for patients with PC following radical prostatectomy.

## 2 PATIENTS AND METHODS

We conducted a cross‐sectional qualitative study investigating stakeholders' perceptions about an mHealth application for PC follow‐up after radical prostatectomy. This qualitative investigation is part of a larger prospective PC registry, ProSquare (NCT04694924), which aims to investigate various aspects of PC management. Ethical approval for this study was obtained through the KU Leuven/UZ Leuven Ethics Committee (MP018039/MP018158). All participants gave informed consent. The consolidated criteria for reporting qualitative research checklist was used to guide reporting (Appendix S3).

Semistructured interviews were conducted in urologists, nurses, and patients face‐to‐face by two researchers (LS and LW). Patient participants were contacted via telephone prior to their scheduled follow‐up visit at the University Hospitals Leuven after a radical prostatectomy. During this phone call, the purpose of the study was explained, and patients were invited to participate in the interview following their hospital visit.

Urologists and nurses who are working in the participating sites of the ProSquare registry (UHL, AZ Groeninge Kortrijk, Sint‐Blasius Dendermonde, and AZ Sint‐Jan Bruges) were asked to provide insights into their experiences and perceptions related to the use of mHealth applications for PC follow‐up. Some of these interviews were conducted online, via Microsoft Teams, to avoid travel between different study sites.

A research team including a urologist, an epidemiologist, and researchers in PC care constructed and piloted the interview guide (Appendices S1 and S2). The interview guide was developed informed by the literature and knowledge of the research team. It was piloted with a urologist to ensure its appropriateness and effectiveness in capturing the desired data. The interviews started with exploratory questions, inquiring about stakeholders' initial perceptions about mHealth applications. Next, the usefulness of an mHealth app to follow‐up patients with PC was questioned. Finally, we inquired about the necessary features of an ideal mHealth app. The duration of the interviews varied between 20 and 30 min. Interviews were conducted in Dutch, audio‐recorded, transcribed ad verbatim, and anonymised for analysis. Only the interviewer and the participant were present during the interview. No repeat interviews took place, and the transcribed interview was not sent back to the participant. Field notes were taken during the interview. Interviews were performed until data saturation was reached. This refers to the point at which little or no relevant new elements were found in data and when issues begin to be repeated with no further understanding or contributions.^19^

The transcripts were analysed and coded by two independent researchers (LS and LW) in accordance with the Qualitative Analysis Guide of Leuven (QUAGOL) method, which is based on the constant comparative method of grounded theory.^20^ Coding and analysis were supervised by two experienced researchers (DDC and AC). Via QUAGOL, themes and subthemes on perceived benefits and barriers for the implementation of mHealth applications for the follow‐up of patients with PC after radical prostatectomy were formed. Additionally, recommended features of an mHealth application were grouped in a separate framework, based on persuasive principles. Persuasive principles are design techniques that motivate users of systems such as mHealth applications. We used the Persuasive Systems Design (PSD) model. PSD consists of 28 persuasive design principles, divided into four categories: Primary Task Support, Dialogue Support, Social Support, and System Credibility.^21^, ^22^, ^23^, ^24^

## 3 RESULTS

A total of 30 stakeholders, including nurses (*n* = 7), urologists (*n* = 8), and patients (*n* = 15), were interviewed between February and April 2022. Benefits and barriers to implement an mHealth application for the follow‐up of PC patients were identified among all stakeholders and grouped into five overarching themes: healthcare optimalisation, disease management, app compliance, legal and organisational requirements, and patient–mHealth interaction (Table 1). Additionally, recommended features for an ideal app were grouped according to the PSD model (Table 2).

**TABLE 1:** Expected benefits and barriers of a mobile health application for the follow‐up of prostate cancer patients, according to urologists (U), nurses (N), and patients (P).

Benefits	Barriers
Healthcare optimalisation Reducing unnecessary consultations (U, N, and P) Structuring of data (U, N, and P) Suggestions for further follow‐up (U) Patient–caregiver interaction (U and N) Primary–secondary care interaction (U and N) Disease management Visualising disease evolution (U, N, and P) Objective follow‐up (U) Recognising individual needs and expectations (U and N) Treatment advice (U)	App compliance mHealth experience (U, N, and P) Questionnaires compliance (U, N, and P) Need for instructions and guidance (U, N, and P) Legal and organisational requirements Validation (U and P) Financial compensation (U and N) Privacy concerns (N and P) Patient–mHealth interaction Trust in mHealth care (P) Telemonitoring versus standard care (P)

**TABLE 2:** Features of a mobile health application for the follow‐up of prostate cancer patients according to stakeholders, grouped by the Persuasive Systems Design model.

	U	N	P
Primary task support Reduction unnecessary consultations Automated processes Structuring of data Personalised data entry Self‐configuration Instructions and guidance Visualising disease evolution Overruling	**X** **X** **X** **X** **X** **X** **X**	**X** **X** **X** **X** **X**	**X** **X** **X** **X**
Dialogue support Reminders for data entry Alerts in case of red flags Social role Suggestions of actions in case of red flags Disease‐related information	**X** **X** **X** **X**	**X** **X** **X**	**X** **X** **X** **X** **X**
Social support Direct communication with healthcare providers Free‐text box Miction diary In between patient comparison Patient–patient interaction Recognising individual needs and expectations	**X** **X** **X** **X**	**X** **X** **X** **X**	**X** **X** **X**
System credibility Application reliability Objective measurement instrument Integration with electronic medical patient files Integration with hospital application In dialogue with healthcare providers Validation	**X** **X** **X** **X** **X** **X**	**X** **X** **X** **X**	**X** **X**

Abbreviations: N, nurses; P, patients; U, urologists.

### 3.1 Benefits

#### 3.1.1 Healthcare optimalisation

##### Reducing unnecessary consultations

All stakeholders believed an mHealth application in clinical practice could reduce unnecessary consultations at the urology department. All stakeholders expected that the app could determine which patients needed urgent care and be prioritised for consultation and which patients needed mHealth follow‐up.

I went in there just now, 10 minutes, my PSA was good, I only had questions on erection, …, but in fact, they didn't check or do anything, so they might as well do that via app or phone. (Patient 12)

Because that will effectively reduce the workload of patients or doctors and will bring us back to the essentials. Where we put more of our time into necessary consultations and much less into useless consultations. So if mHealth can help, which I do think it can, then I definitely support that. (Urologist 5)

I think that will add value for a large proportion of patients. Especially for men who are going well now. And who actually, to say in their spare time need to come here to just say my PSA is good, urination is going well, erectile function is going well. (Nurse 2)

Patients emphasised that less unnecessary consultations can lower travel‐time and cost. Comparisons were made between the local journey to a general practitioner (GP) while visiting the hospital is a longer journey.

If I go to the GP, I can go there on foot, but with this, the hospital is far away, always a risk, fuel …. (Patient 12)

##### Structuring of data

All stakeholders stressed that an mHealth application can optimise the structure of the medical record, allowing functional and oncological data to be retrieved more efficiently. Healthcare providers also expected that a better data structure lowers the time of a consultation.

I sometimes have to spend a long time looking for that in the file. It would be handy if you can quickly look that up in the app. (Urologist 2)

If that app of yours does that [collecting the PSA‐values], then it's going to help a lot of people who didn't, like me, and the urologist will start getting more oversight. (Patient 14)

I think then we will overlook fewer things and then also start talking more deeply in certain areas that he wouldn't otherwise say to the doctor. (Nurse 2)

Patients expressed that the app could give a clear overview of data and availability of clinical results.

If that app of yours does that [collecting the PSA‐values], then it's going to help a lot of people who didn't, like me, and the urologist will start getting more oversight. (Patient 14)

##### Suggestions for further follow‐up

Urologists expected that an mHealth application could utilise questionnaires to identify the most prominent functional complaints after PC treatment. This could determine the choice of physician, depending on the physician's specialisation, or even a particular care pathway.

So, when the patient sees that he has bladder problems he comes to see me. And if the patient has problems with his PSA‐level he comes to see Professor X. That, of course, would be the ultimate goal. (Urologist 1)

##### Patient–caregiver interaction

Healthcare providers emphasised that reviewing the data collected by an mHealth application between clinic visits could improve communication with patients and allow sensitive functional complaints to be addressed more smoothly.

When it's a topic like erectile dysfunction. That is such a subject that is not easily brought up and you sometimes want to put your time into it. But it's one of those questions among the 100 questions that you ask during the consultation and then go over too quickly. The patient then goes home with little answer and if it then turns out in the app that that's the thing that causes the most problems for the patient. (Nurse 2)

So that the patient who comes for interim check‐ups also fills in those questionnaires. Then I can assess their status a bit better. That saves a lot of questions for me as well. Then you can get straight to the point. (Urologist 5)

##### Primary–secondary care interaction

Healthcare providers expected that the application could improve communication between primary and secondary care by reducing the GP's workload and by lowering the risk of a late referral to the hospital.

Because what is my experience with GP's. Many kinds of people in there. So I see that GPs sometimes can't get away with it and don't have enough knowledge in that to know: “I have to refer that one.” (Nurse 6)

We actually say now if the PSA is good, they have an unmeasurable PSA and they have no functional complaints. Then we now say follow‐up through the GP twice a year via blood sampling. But very occasionally we then do see that the patient is referred back to us too late. (Urologist 3)

#### 3.1.2 Disease management

##### Visualising disease evolution

All stakeholders considered the visualisation of patients' disease evolution both in terms of PSA variability and functional complaints as a crucial benefit.

Now we get lab results and then you have to go back in the file and look for what is the previous figure and then you don't see the evolution. (Nurse 1)

You can remember that it [PSA value] was normal and suddenly it's abnormal, but yes the easiest thing is from just having a curve to see yes it stays flat or it goes up. (Patient 7)

So that when the patient comes for a consultation you quickly see an overview of what the main problems are and that you can also see an evolution. (Urologist 2)

##### Objective follow‐up

Urologists mentioned the benefits of measuring PSA in the app as it is an objective indication of a patient's oncological status and easily interpreted by both urologists and patients.

Because you have a quantitative value there, the PSA that you can easily telemonitor and that patients can also easily interpret. (Urologist 7)

##### Individual needs and expectations

Nurses and urologists perceived that by tracking the functional complaints via questionnaires, patient's needs and expectations could be more efficiently identified and addressed in practice.

In addition to the questionnaire, I would also ask the patient: do you want something done about those complaints? Because some patients have complaints but consciously choose not to get treated for it. (Urologist 6)

I think we then overlook fewer things and then also talk more deeply in certain areas that he would not otherwise say to the doctor. (Nurse 2)

##### Treatment advice

Urologist felt the app could personalise patients' advice. By benchmarking patients, the patient's specific status could be better framed within the population and aid in treatment choice. Urologists believed that the app would increase a patient's understanding of his complaints and lead to a more informed shared decision.

I would also find it interesting to be able to compare that score with the average score. To see whether that one [patient] does better or worse. (Urologist 3)

### 3.2 Barriers

#### 3.2.1 App compliance

##### mHealth experience

Being less digitally experienced was perceived as a barrier towards the use of mHealth applications by all stakeholders, especially in a population of older age.

But actually I don't have an app, or I don't use that but my wife has a mobile phone and possibly I can do it with that. (Patient 5)

If we ask the over‐80s are you accessible via the internet, the answer is often: “I don't need to know anything about the internet.” So the smartphone apps they are not all going to be ready for that now. In time they will be, of course. (Nurse 4)

Because we also work with a patient population that is slightly older. But of course there is a shift coming now. The guys who are now 50, 55, they get already more along with applications and so on. (Urologist 2)

##### Questionnaires compliance

Moreover, all stakeholders expressed concerns about using extended questionnaires, to monitor functional complaints and quality of life. They expected that adherence would decrease over time.

I wouldn't make that [the questionnaire] longer than 10 minutes. (Patient 13)

I think that are two good questionnaires but long questionnaires. My experience is that long questionnaires put people off. (Urologist 3)

Yes, because I think if they keep having to fill in those lists always that they're going to be like, there they are with all their things again. (Nurse 7)

##### Need for instructions and guidance

All stakeholders stressed the importance of education to learn patients how to use an mHealth application.

It would be perfect, the moment you put a patient on the app and come to the onco nurses, that they take a moment to go over there and explain: how does this app work. (Urologist 8)

So I think if there is a good app that is user‐friendly and regularly referred to and indicated by us, the GPs the specialists that it will be used by patients. (Nurse 1)

For me it is more difficult, if there is something with the computer or so I always have to go to the children. (Patient 12)

#### 3.2.2 Legal and organisational requirements

##### Validation

Patients emphasised their preference for manual validation by a healthcare professional of each mHealth report. Time between unfavourable results and an actual contact moment needed to be minimised. In contrast, urologists were worried that manual validation of each report would increase workload. They also questioned how accurately each report can be reviewed by a physician and believed a filter could lower workload.

There is no doctor who reviews 100 reports very carefully. But if there is a system where filtering is done. Then that can also be a strict system with a low threshold that is going to say “you have to check that, check that.” But if you then already have a 50% reduction in the number of reports, then that is useful. (Urologist 6)

Yes, [a message by a healthcare professional stating] like “okay, we have seen it and effectively it is okay.” (Patient 4)

##### Financial compensation

Healthcare providers were concerned that an mHealth application would significantly increase the amount of patient data to be processed, consequently increasing their workload. They felt that an appropriate legal framework concerning financial compensation should be established to enable mHealth‐guided care.

That from the government it is said, look if you use this system then we are going to score a lot of profit on care that shouldn't be delivered but then you do get a compensation. (Urologist 6)

Uhm, I think the doctor is going to want that. Here in the hospital that's his right, he's not on a fixed amount here or anything. So they get paid per performance. (Nurse 5)

##### Privacy concerns

Both patients and nurses were concerned about the security of patients' health information. They emphasised the need for privacy rules concerning health information.

Of course it does matter that there are privacy rules and that things cannot be hacked. (Nurse 4)

I could give you a whole lecture on that, I think there is already too much sharing of information today, but concerning the app, I would like to limit parts of the info send to the hospital, the GP and the patient themselves. (Patient 13)

#### 3.2.3 Patient–mHealth interaction

##### Trust in mHealth care

Patients expressed concerns about the accuracy of mHealth monitoring. Additionally, patients strongly emphasised that an mHealth application should be used as a decision‐support tool and never replace genuine social interaction.

Well now, easy to fill in yes, but is it clear for the person who has to analyse and interpret it? (Patient 8)

##### Telemonitoring versus standard care

Some patients expressed a preference for standard care rather than mHealth monitoring to better convey the importance of a particular complaint to the healthcare provider during physical consultations.

Hmm, for me personally I do prefer to have some contact with a professor or an assistant, then one can still communicate a bit themselves and this can make me feel more safe …. (Patient 1)

### 3.3 Recommended features of an mHealth application

Table 2 summarises the recommended features, based on stakeholders' suggestions, for an ideal mHealth application in PC follow‐up of according to the PSD model.

#### 3.3.1 Primary task support

Reducing the number of non‐essential consultations was prioritised by all stakeholders. Urologists and nurses indicated that this process ideally should be automated. Furthermore, the application should structure the medical record to retrieve data. Urologists indicated that the content of questions could be personalised or self‐configured per patient, by including the possibility to accept a teleconsultation or by setting PSA limits. Healthcare providers stressed the importance of getting the patient started in the use of the app, by using a QR code or instructions manuals. All stakeholders expected an improvement of visual or graphical feedback of disease status. Most urologists would present PSA values over time. Some urologists indicated that functional complaints should be graphed per domain and over time. Some nurses suggested ordering data, with answers with higher medical needs being placed on top. Furthermore, patients indicated that an option to overrule the mHealth app despite a favourable outcome could be of added value.

#### 3.3.2 Dialogue support

Stakeholders mentioned reminders when data entry is due. Moreover, an alert was needed when PSA values reach a threshold or functional complaints were detected. Participants mentioned the hospital's responsibility of contacting patients when PSA levels reach a threshold. Urologists suggested that the app could make appointments to the appropriate specialist in case of functional level changes. Patients indicated colour coding to highlight results together with an understandable explanation in a physician's report and optionally additional disease‐related information.

#### 3.3.3 Social support

Direct communication with their healthcare provider was considered by stakeholders as an important functionality. The use of a free‐text box and miction diary were suggestions to encourage communication. The ability to benchmark patients was also cited by urologists to be implemented in the application. Some patients suggested a social feature in the app to connect with other patients.

#### 3.3.4 System credibility

Stakeholders indicated that the application should be reliable and that the app content should be based on validated disease status measures. Urologists strongly emphasised using PSA as an objective measurement tool. Care providers wanted the mHealth application to be integrated within the medical patient files or linked to their hospital application. They stated that the application needed to be created in dialogue with healthcare providers.

## 4 DISCUSSION

We investigated the perceptions and recommendations of stakeholders on mHealth in the follow‐up for patients after radical prostatectomy. All stakeholders expected benefits in care optimisation and disease management by reducing unnecessary clinic visits and allowing more attention and time for patients with adverse oncological or functional outcomes.

Urologists highlighted that monitoring oncological status should be straightforward with mHealth using serum PSA as a reliable postoperative biomarker. Nurses emphasised the importance of following‐up functional outcomes after radical prostatectomy and the impact on patient's quality of life. Although the use of patient‐reported outcome measures (PROMs) has emerged as a valuable tool in assessing functional outcomes following radical prostatectomy,^25^ most healthcare providers argued in our study that long questionnaires should be avoided to prevent patient burden and data quality concerns. Patients were willing to receive care using digital technologies, such as mHealth, but underlined the need for a simple interface, with minimal but personalised input. Previous studies investigating other technologies, such as videoconferencing, confirmed a high acceptance for tailored post‐surgical care in PC survivors.^26^

Several challenges have been identified in the follow‐up of PC survivors: (a) Patients do not know not how to interpret their functional outcomes and how they compare with other PC patients; (b) healthcare providers are not optimally informed about the specific problems of each individual patient; and (c) patients receive fragmented care due to a lack of collaboration between different healthcare providers and/or healthcare organisations.^4^, ^27^, ^28^, ^29^, ^30^ All stakeholders in our study were convinced that an mHealth application can constitute a solution for these challenges. (a) By offering a visual overview of the individual's progression of a patient's functional and biochemical outcomes, the treating healthcare provider as well as the patient himself would gain more insight into his disease activity. Additionally, urologists felt that the app could personalise patients' advice. By benchmarking patients, the patient's specific status could be better framed within the population and aid in treatment choice. (b) Healthcare providers mentioned adding specific questions to the questionnaire, such as “do you want to be treated for this complaint” and using a free‐text box, which will help identifying and addressing patient's individual needs and expectations in practice. Studies show that the possibility of being able to discuss the remotely collected information during a consultation is appreciated by the patient and can contribute to an improved patient–caregiver interaction.^31^, ^32^ (c) The mHealth application could ameliorate communication between primary–secondary and tertiary care by decreasing GP's workload, by improving the interpretation of the PSA score, by providing an alert when the PSA score reaches the threshold, and by avoiding critical situations such as late referral of patients to second‐line care.

In addition to perceived benefits and barriers for the implementation of mHealth apps, study participants also identified a number of features the ideal mHealth app should offer. To examine the expected impact of these features on user engagement, we divided proposed features into four categories according to the PSD model. Features mentioned by all stakeholders were as follows: reduction of unnecessary consultations, automated processes, visualisation disease evolution, reminders for data entry, alerts in case of red flags, social role, direct communication with healthcare providers, free‐text box, reliability of the application, and validation. A previous study investigated the applicability of the Rhythm Prostate application for PC survivors on three core functionalities: dynamic visualisation, intelligent reminders, and direct communication. The majority of participants agreed that the three core functionalities of Rhythm Prostate were useful to them (20/28, 71% [dynamic visualisation]; 16/28, 57% [intelligent reminders]; and 18/28, 64% [direct communication]).^33^ These features also recurred during our interviews and are therefore best implemented in the application.

Our study has several strengths and limitations. First, participants were individually interviewed, and not in focus groups. Focus groups might encourage more interaction between participants to elicit more information yet some participants might feel less comfortable in group.^34^ Second, our interviews were limited to patients, urologist, and PC nurses. Other healthcare providers including GPs and oncologists, government representatives, app developers, and psychologists might have also provided valuable insights. Third, although the interviews with physicians and nurses were conducted in diverse settings in Flanders, representing both academic and general hospitals, the 15 participating patients were in follow‐up at a tertiary centre. This convenience sampling method may cause a selection bias that lowers the generalisability for all patients with PC after prostatectomy. Fourth, most study participants had limited experience with mHealth applications, which reduced the risk of strongly opinionated. Our results should therefore not be interpreted as experience‐based suggestions for improvement. Nevertheless, this “app‐naïve” study population could serve as a blueprint to develop the optimal mHealth ‐app. Finally, the robustness of our findings was improved by analysing the results with an interdisciplinary research team, guided by the QUAGOL.

## 5 CONCLUSIONS

Stakeholders including patients, urologists, and urology nurses expected that an mHealth app in the care for patients with PC following radical prostatectomy might optimise care and promote disease management of PC but were concerned that app compliance would be a challenge.

## AUTHOR CONTRIBUTIONS

*Conceptualization*: Diederik De Cock and Wouter Everaerts. Methodology: Diederik De Cock, Lucas Schreurs, Lars Wouters, Andries Clinckaert, and Wouter Everaerts. *Writing—original draft preparation*: Andries Clinckaert, Lucas Schreurs, Lars Wouters, and Diederik De Cock. *Writing—review and editing*: Andries Clinckaert, Lucas Schreurs, Wouter Everaerts, and Diederik De Cock. *Visualization*: Andries Clinckaert and Lucas Schreurs. *Supervision*: Wouter Everaerts and Diederik De Cock. All authors have read and agreed to the published version of the manuscript.

## CONFLICTS OF INTEREST

The authors declare no conflict of interest.

## Supporting information


**Appendix S1** Interview guide part A

**Appendix S2** Interview guide part B

**Appendix S3** ISSM_COREQ_Checklist
