# Supplementary material for: “Keep It Short and Simple”: Perceptions of patients and healthcare professionals on the use of a mobile health app in the care for patients undergoing radical prostatectomy
Source: BJUI Compass. 2023 Jul 15;5(1):150–8. doi: 10.1002/bco2.270 (PMC10764175; doi:10.1002/bco2.270)
Supplement: Supplementary file 1 — Appendix S1 Interview guide part A [file BCO2-5-150-s002.pdf]

Appendix I: interviewguide

INTERVIEWGUIDE VOOR

DE HINDERPALEN EN FACILITERENDE FACTOREN BIJ ZORGVERLENERS VOOR HET GEBRUIK VAN EEN MOBIELE HEALTH APP IN DE OPVOLGING VAN PATIËNTEN NA PROSTAATKANKERCHIRURGIE.

Via een kwalitatieve studie wordt de mening van zorgverleners bevraagd over het gebruik van een mobiele health app in de opvolging van prostaatkankerchirurgie.

INLEIDENDE VRAGEN:

Wat is uw mening over het gebruik van mHealth in de opvolging van patiënten? Denkt/weet u of uw patiënten mHealth toepassingen reeds gebruiken?

TOPIC VRAGEN:

In welke mate denkt u dat een gezondheidsapp bruikbaar kan zijn voor de opvolging van uw prostaatkankerpatiënten?

Vindt u de vragenlijsten (EPIC 26 en QLQ C30) in verband met prostaatkanker voldoende om te gebruiken in de app?

Mogelijke bijvragen als deze punten niet ter sprake komen:

- Dekken de vragenlijsten voldoende de lading?
- Welke topics moeten nog bevraagd worden volgens u?
- Hoelang moet deze opvolging duren?
- Denkt u dat het gebruik van een gezondheidsapp een impact zal hebben op de werklast?
- In welke mate denkt u dat deze mHealth app een meerwaarde kan bieden naast de standaard zorg?

Welke voorwaarden moet deze app bieden vooraleer u (als zorgverlener) zou overwegen om deze te gebruiken?

Mogelijke bijvragen als deze punten niet ter sprake komen:

- Moet er manuele validatie zijn van elk mHealth? rapport en zo ja wie mag/kan dit doen?
- Wie mag de resultaten zien? (huisarts /onderzoekers)
- Moet een financiële vergoeding voorzien worden?

Hoe wenst u feedback over de vragenlijsten te geven/krijgen?

Mogelijke bijvragen als deze punten niet ter sprake komen:

- Hoe wilt u de resultaten weergeven? (grafiek, persoonlijk rapport, telefonisch)
- Hoe wilt u contact opnemen indien er nood is aan verdere opvolging (zelf bellen, patiënt initiatief laten nemen)
- Hoe wilt u een overzicht krijgen van de bekomen resultaten van de app.
