# Supplementary material for: “Keep It Short and Simple”: Perceptions of patients and healthcare professionals on the use of a mobile health app in the care for patients undergoing radical prostatectomy
Source: BJUI Compass. 2023 Jul 15;5(1):150–8. doi: 10.1002/bco2.270 (PMC10764175; doi:10.1002/bco2.270)
Supplement: Supplementary file 2 — Appendix S2 Interview guide part B [file BCO2-5-150-s001.pdf]

8. Bijlagen

8.1 Bijlage I: het semi-gestructureerde interview

Interviewguide voor:
De faciliterende factoren en hinderpalen voor het gebruik van een mobile health app voor opvolging van prostaatkanker patiënten

Via deze kwalitatieve studie worden relevante stakeholders bevraagd afkomstig uit de regio Vlaanderen. Deze stakeholders zijn patiënten en patiëntvertegenwoordigers. In dit document wordt de interviewguide voorgesteld.

- **Inleidende vragen**
  - ❖ Heeft u ooit al gebruik gemaakt van een gezondheidsapp?
    - ➢ Indien ja; Welke app heeft u dan al eens gebruikt / gebruikt u momenteel?
      Wat vond u van deze app?
      Waarom gebruikt u deze app?
      Hoe lang gebruikt u deze app al?
    - ➢ Indien nee; korte uitleg.
  - ❖ Heeft u al gebruik gemaakt van MyNexuzHealth?

- **Topicvragen**
  - ❖ In welke mate denkt u dat een gezondheidsapp bruikbaar / nuttig kan zijn voor **de opvolging** van uw prostaatkanker?
    - o Vragenlijsten en PSA (meting via de huisarts) worden ingegeven door patiënt
      - ▪ Vindt u de vragenlijsten (van net voor de operatie) in verband met prostaatkanker voldoende om te gebruiken in de app?
        - In welke mate peilen deze vragen naar relevante onderwerpen voor u?
        - Welke andere vragen ontbreken volgens u in deze lijst?
      - ▪ Frequentie van opvolging/vragenlijsten.
        - Hoelang vindt u dat deze opvolging via een mobile health app kan duren?
    - o Standaard opvolging vervangen (Uroloog zien in het ziekenhuis.)
      - ▪ In hoeverre denkt u dat de vragenlijsten + PSA de standaard opvolging kunnen vervangen?
  - ❖ Welke “voorwaarden” moet deze app bieden vooraleer u zou overwegen deze te gebruiken?
    - o Wanneer zou u deze app als “gebruiksvriendelijk” beschouwen?
    - o In welke mate moet er automatisering zijn in de app?
      (Eventueel korte uitleg over automatisering.)
      Wat is uw mening over een optie tot “overrulen” of om toch contact aan te vragen?
      (Eventueel korte uitleg over “overrulen”.)
      Heeft u enige bedenkingen met betrekking tot privacy?
      (Wie mag resultaten zien? Delen met huisarts / onderzoekers / …)
  - ❖ Hoe wenst u feedback over uw vragenlijsten te horen/krijgen?
    - o Welke manier van communicatie? (grafiek, persoonlijk rapport, telefonisch)
    - o Hoe snel wenst u feedback te krijgen?
    - o Hoe wilt u contact opnemen indien “slecht” nieuws?
      (Zelf bellen, patiënt initiatief laten nemen, gebeld door arts…?)
